# Parcellation of Human and Monkey Core Auditory Cortex with fMRI Pattern Classification and Objective Detection of Tonotopic Gradient Reversals

**DOI:** 10.1093/cercor/bhu124

**Published:** 2014-06-05

**Authors:** Marc Schönwiesner, Peter Dechent, Dirk Voit, Christopher I. Petkov, Katrin Krumbholz

**Affiliations:** 1 Laboratory for Brain, Music and Sound Research (BRAMS), Montreal, Canada; 2 Department of Psychology, University of Montreal, Montreal, Canada; 3 Montreal Neurological Institute, McGill University, Montreal, Canada; 4 Department of Cognitive Neurology, MR-Research in Neurology and Psychiatry,University Medicine Göttingen, Göttingen, Germany; 5 Biomedical NMR Research GmbH, Max-Planck-Institute for Biophysical Chemistry, Göttingen, Germany; 6 Institute of Neuroscience, Newcastle University Medical School, Newcastle upon Tyne, UK; 7 MRC Institute of Hearing Research, Nottingham, UK

**Keywords:** hearing, tonotopy

## Abstract

Auditory cortex (AC) contains several primary-like, or “core,” fields, which receive thalamic input and project to non-primary “belt” fields. In humans, the organization and layout of core and belt auditory fields are still poorly understood, and most auditory neuroimaging studies rely on macroanatomical criteria, rather than functional localization of distinct fields. A myeloarchitectonic method has been suggested recently for distinguishing between core and belt fields in humans (Dick F, Tierney AT, Lutti A, Josephs O, Sereno MI, Weiskopf N. 2012. In vivo functional and myeloarchitectonic mapping of human primary auditory areas. J Neurosci. 32:16095–16105). We propose a marker for core AC based directly on functional magnetic resonance imaging (fMRI) data and pattern classification. We show that a portion of AC in Heschl's gyrus classifies sound frequency more accurately than other regions in AC. Using fMRI data from macaques, we validate that the region where frequency classification performance is significantly above chance overlaps core auditory fields, predominantly A1. Within this region, we measure tonotopic gradients and estimate the locations of the human homologues of the core auditory subfields A1 and R. Our results provide a functional rather than anatomical localizer for core AC. We posit that inter-individual variability in the layout of core AC might explain disagreements between results from previous neuroimaging and cytological studies.

## Introduction

Anatomical and neurophysiological studies have established a widely accepted model of the structure of auditory cortex (AC) in non-human primates (Merzenich and Brugge 1973; Imig et al. 1977; Morel and Kaas 1992; Morel et al. 1993; Rauschecker et al. 1995; Hackett et al. 1998; Kaas and Hackett 1998, 2000). According to this model, primate AC contains 3 primary-like fields, collectively referred to as “core,” each with a separate map of sound frequency (tonotopic map). In the macaque, the core fields are stacked in a posterior-to-anterior direction and surrounded by secondary fields, referred to as “belt” (reviewed in Petkov et al. 2006; Baumann et al. 2013). Cytoarchitectonic data from postmortem human brains are consistent with this model of primate AC: they suggest that human AC also contains 3 primary-like fields, stacked along the long axis of Heschl's gyrus (HG), which runs in a posteromedial-to-anterolateral direction (Morosan et al. 2001). Functional imaging data, however, have sometimes led to conflicting interpretations (reviewed in Baumann et al. 2013). Most functional imaging studies found evidence for 2 primary-like fields, which were interpreted as the human homologues of A1 and R; rarely has evidence for a third primary-like field (RT) been found (see, however, Moerel et al. 2012). However, different studies have reached conflicting conclusions about the fields' orientations and tonotopic layouts with respect to macroanatomical landmarks (reviewed in Baumann et al. 2013). All studies have used tonotopic mapping to detect borders between fields within core AC. The main difference between studies that have reached conflicting conclusions is that some studies have used an independent criterion for distinguishing between core and belt auditory fields. In visual cortex, the boundary between the primary field (V1) and non-primary fields is marked by reversals in the polar-angle component of the retinotopic gradient (Engel et al. 1994; Sereno et al. 1995). Data from non-human primates and other animals demonstrate that, in AC, the borders between different core fields run perpendicular to the fields' tonotopic gradients and are thus marked by gradient reversals. In contrast, the borders between core and belt fields run parallel to the tonotopic gradients. This means that these borders are not associated with gradient reversals (Morel et al. 1993; Rauschecker et al. 1995; Petkov et al. 2006) and can thus not be detected by tonotopic mapping. Dick et al. (2012) circumvented this problem by using a myeloarchitectonic marker for core AC and then restricting the tonotopic mapping to the core fields so identified. Building upon earlier findings of higher myelination in core fields (Hackett et al. 2001), they improved a previously developed quantitative T1 mapping protocol (Sigalovsky et al. 2006) to estimate myelination across the cortical surface. This method highlighted a region of high myelination on medial HG, consistent with the location of core AC in cytoarchitectonic parcellations in humans (Morosan et al. 2001). The region contained 2 tonotopic gradients oriented along the same axis as observed in monkeys (Merzenich and Brugge 1973; Imig et al. 1977; Morel and Kaas 1992; Morel et al. 1993). Moerel and colleagues found similar results using the independent functional criterion of frequency selectivity, which has been shown to be greater in core than belt fields (Rauschecker et al. 1995).

In the present study, we propose an independent approach to localize core AC based on functional rather than anatomical properties. Most previous attempts at localizing core AC from functional data (e.g., Wessinger et al. 2001; Petkov et al. 2006; Chevillet et al. 2011; Moerel et al. 2012) exploited the finding from neurophysiological studies in monkeys that core AC responds better, and with greater frequency specificity, to pure tones and other narrowband sounds than belt AC (Morel et al. 1993; Rauschecker et al. 1995). The current approach is based on the same general idea but uses multi-voxel pattern classification and high-resolution fMRI to maximize sensitivity to the functional differences between core and belt AC. We independently validate our method by demonstrating that it correctly identifies core auditory fields in several macaque monkey fMRI datasets. We then measure tonotopic gradients to delineate the borders of the subdivisions of core AC. Previous studies have assessed tonotopic gradients by manually connecting their low- and high-frequency endpoints, which involves a degree of subjective interpretation. Moreover, some of the endpoints used may have been part of belt AC, which may have skewed the gradient orientations. Here, we restrict the measurement of tonotopic gradients to the region identified as core by the pattern classification procedure, and we use an automated, and thus objective, method for assessing gradient orientations. We use these methods to delineate subdivisions of core AC in several individuals.

## Materials and Methods

### Participants

Seven human participants (4 males, aged between 25 and 35 years) took part in the experiment after having provided informed consent. All were right-handed according to the Edinburgh inventory (Oldfield 1971) and had no history of hearing disorder or neurological disease. The experimental procedures conformed to the World Medical Association's Declaration of Helsinki and were approved by the local ethics committee.

In addition, this paper also presents fMRI datasets from 3 macaque monkeys. One monkey (Monkey 1) was scanned while anesthetized, the other 2 (Monkeys 2 and 3) were awake. Two of the monkey datasets (Monkeys 1 and 2) were acquired at the Max-Planck-Institute of Biological Cybernetics, Germany (Logothetis group) and have been used in a previous study (Monkeys 1 and 2; Petkov et al. 2006). The third dataset (Monkey 3) is new and was acquired at Newcastle University, United Kingdom (Petkov group). Procedures for animal handling, anesthesia, and scanning complied with the guidelines of the European Community (EUVD 86/609/EEC) for the care and use of laboratory animals and were approved by the local authorities. A detailed description of all procedures can be found elsewhere (Logothetis et al. 1999; Petkov et al. 2006).

### Stimuli

For the human measurements, the sound stimuli consisted of bursts of pure tones with frequencies centered around 1 of 8 nominal frequencies, logarithmically spaced between 200 and 8000 Hz (200, 338.8, 573.8, 971.9, 1646.2, 2788.4, 4723.1 and 8000 Hz). The bursts were 187.5 ms in duration (including 20-ms squared-cosine onset and offset ramps) and presented once every 250 ms for 4 s. The stimuli were generated digitally (24.4-kHz sampling rate, 24-bit amplitude resolution) using Matlab and Tucker Davis Technologies System 3. In order to minimize response adaptation, the frequency of the tone bursts was varied randomly according to a uniform distribution of 1 semitone around the nominal frequency. These frequency variations were large enough to be audible, but, at the same time, small enough to be processed within the same cochlear channel (Glasberg and Moore 1990). The stimuli were presented binaurally at a level of 75 dB SPL through MR-compatible high-fidelity headphones (MR Confon).

In order to minimize the effect of the variation in the normal hearing threshold across frequency, as well as differences in hearing threshold between participants and inhomogeneities in the headphone transfer function, the stimuli were presented in a background of continuous noise with equal energy per cochlear filter bandwidth (defined as equivalent rectangular bandwidth, or ERB; Glasberg and Moore 1990) across all frequencies. The noise was presented throughout the experiment at a level of 35 dB SPL per ERB, 40 dB below the level of the tones.

Two of the 3 monkeys were presented with 6 pure tones, logarithmically spaced in frequency between 500 Hz and 16 kHz. The third monkey was presented with 2 2-octave-wide noises, centered at 500 Hz and 4 kHz. The stimuli were generated digitally with a 44.1-kHz sampling rate. They had a 50-ms duration (including 8-ms raised-cosine onset and offset ramps) and were presented with a 75-ms inter-stimulus interval and at an intensity of 75 dB SPL. They were presented through MR-compatible headphones modified to fit the monkeys (MR Confon, for Monkeys 1 and 2, and NordicNeuroLab, for Monkey 3).

### Procedure

In the human measurements, each trial started with the presentation of a stimulus for 4 s, followed by the functional image acquisition, which took 1 s and then 5 s of silence to yield a 9-s repetition time (TR) in order for any activation due to the scanner noise to die away before the next image acquisition. The stimuli were presented in epochs consisting of 2 trials, followed by 2 baseline trials where the stimulus was replaced by 4 s of silence. The experiment contained 20 trials of each of the 8 frequencies (see above). Epochs for different frequencies were presented in pseudorandom order with balanced transition probabilities. The experiment consisted of 324 trials in total (20 trials × 8 frequencies + 160 baseline trials + 4 initial dummy trials). It was split evenly into 2 runs of equal duration separated by a short break. Together with the structural scans, each session lasted about 60 min. This was a passive listening experiment; in order to stay alert, participants watched a silent subtitled movie of their own choice, which was presented through a projection system with vision-correcting goggles. While it has been shown that task context can have a strong influence on AC activity (reviewed in Fritz et al. 2007), there is evidence that attention has little effect on measures of tonotopic organization (Petkov et al. 2004; Woods et al. 2009).

In the monkey measurements, each trial started with the presentation of a train of stimuli for 4–8.5 s, followed by the functional image acquisition, which took 1.5 s to yield a 10-s repetition time (TR; see Petkov et al. 2006 and Petkov et al. 2009 for details). Trials containing sounds were alternated with silent baseline trials. The awaken monkeys were required to maintain visual fixation throughout the auditory stimulation, and so, the number of repetitions per condition, as well as the number of conditions depended on fixation performance. Monkeys 1 and 2 were presented with 12 and 29 repetitions of each of 6 frequencies, respectively, and Monkey 3 was presented with 60 repetitions of the 2 frequency conditions (see above).

### Imaging Protocol

The human measurements were performed in a horizontal 3-Tesla scanner (Magnetom TIM Trio, Siemens Healthcare) equipped with a 12-channel matrix head-coil (Siemens Healthcare) and using an echo-planar imaging sequence (gradient echo; acquisition time = 1 s, echo time = 36 ms; flip angle = 90°) with sparse sampling (TR = 9 s) to minimize the effect of scanner noise on the measured activity (Edmister et al. 1999; Hall et al. 1999). Each functional volume comprised 13 slices with an in-plane resolution of 1.5 × 1.5 mm and a thickness of 2.5 mm. The field of view was 192 mm. The slices were oriented parallel to the average angle of left and right lateral sulci (determined from the structural scan, described later) to fully cover the superior temporal plane (STP) in both hemispheres. As a result, the functional volumes included HG, planum temporale (PT), planum polare, and the superior temporal gyrus and sulcus. A standard whole-brain T1-weighted structural scan (magnetization-prepared rapid gradient echo sequence) with 1-mm^3^ resolution was also obtained for each participant (acquired before the functional scans).

The monkey measurements were performed in vertical primate scanners (Bruker Medical) at 4.7 T (Monkeys 1 and 3) and 7 T (Monkey 2). A primate chair was used for positioning the animals within the magnet bore. Functional and structural volumes were acquired using surface radiofrequency coils of 70- (Monkey 1) or 80-mm (Monkey 2) diameter, positioned over AC of 1 hemisphere, or a whole-head coil (Monkey 3). Only the right AC was measured with the surface coils (Monkeys 1 and 2), whereas both the left and right ACs were measured with the head coil (Monkey 3). Functional volumes were acquired using a gradient-recalled echo-planar imaging sequence (TE = 16 ms, acquisition time = 1.5 s, TR = 10 s). They consisted of 6–12 slices, oriented parallel to the relevant lateral sulcus. Each slice consisted of 128 × 128 voxels and was 2 mm thick. The field of view was set individually for each animal and measured between 6.4 × 6.4 and 12.8 × 12.8 cm, corresponding to an in-plane resolution of between 0.5 × 0.5 and 1 × 1 mm. Structural volumes were acquired with a 3-dimensional, modified driven-equilibrium Fourier transform sequence (TE = 4 ms, TR = 22 ms, 256 × 256 × 128 voxels, with an FOV of 9.6 × 9.6 × 6.4 cm).

### Data Analysis

The functional data from both the humans and the monkeys were corrected for motion artifacts and spatially smoothed with a 2-mm Gaussian kernel. Statistical analysis was based on a general linear model (GLM) implemented in BrainVoyagerQX and performed on the original 3D (“volume”) data. Regions of significant activation were determined by comparing the responses to the sound conditions with the silent baseline. The GLM was applied both to the individual and fixed-effects group data. For the group analysis and for comparison of individual datasets, the functional volumes were co-registered to the symmetric ICBM152 template. Further analyses were performed in Matlab after importing the data using BrainVoyager's Matlab toolbox BVQXtools.

First, we extracted frequency tuning curves for each voxel by plotting the response size (compared with baseline) to the different frequency conditions. In previous studies, the voxels' preferred (or “best”) frequencies have been estimated as the frequency yielding the largest response, or by fitting the voxel frequency tuning curves with a bell-shaped function (e.g., Formisano et al. 2003). The function-fit method has the advantage of utilizing the responses to all frequencies, rather than to just 1 frequency. This reduces susceptibility to noise. However, fitting can become unpredictable when the voxel tuning curves are multi-peaked. Here, we propose a simpler, non-parametric, method, which also utilizes the responses to all frequencies, but without the unpredictability of fitting. Our method involves calculating the centroid of each voxel's tuning curve according to:

$$\text{Centroid}=\frac{1}{n}\sum_{i=1}^{n}\frac{r_{i}}{n\cdot \bar{r}}\cdot f_{i},$$

where n is the number of presented frequencies (8 in the current experiment), $f_{i}$ is the frequency value, and $r_{i}$ is the respective response amplitudes in percent signal change. $\bar{r}$ is the average response amplitude across frequencies. We then estimated the widths of the voxel tuning curves by calculating the spread about their centroids. This involved multiplying the normalized responses, $r_{i}\text{/}(n\cdot \bar{r}),$ with the squared distances of the respective frequencies from the centroid, summing across frequencies, and taking the square root. The tuning-curve spreads were then multiplied by $2\cdot \sqrt{2\cdot \ln\;2}$ to derive the full-width-at-half-maximum (FWHM).

For comparison, we also fitted the voxel tuning curves with a rounded exponential (roex) function (the roex function is bell-shaped and is commonly used for fitting cochlear filters from notched-noise data; Rosen and Baker 1994). The fit was constrained so that the maximum of the function fell in the frequency range covered by the stimuli. The best frequencies and tuning-curve widths were taken as the peak and FWHM of the fitted functions.

The multi-voxel pattern classification analysis of the human data was conducted with a type-2 linear-kernel Support Vector Machine (SVM, Cortes and Vapnik 1995), implemented in the PyMVPA Python toolbox (Hanke et al. 2009), and applied to the volume data of each participant separately. Support vector machines are supervised learning methods that construct 2-category boundaries in sets of training items, each marked as belonging to 1 of the 2 categories. Based on the learned category boundaries, the SVM then predicts whether a new item falls into one category or the other. SVMs only solve binary (2-category) classification problems. Our 8-category frequency classification problem was solved by partitioning into a complete set of pair-wise binary problems with a majority voting strategy to determine the overall classification. This partitioning procedure is part of the PyMVPA toolbox. Prior to the classification analysis, the voxel time series were detrended by robust locally weighted least-squares regression (Cleveland and Devlin 1988), mean-corrected, and normalized by their standard deviation. No spatial smoothing was used in this case. The training and test phases of the SVM analysis were performed using a 5-fold cross-validation procedure, whereby the dataset is randomly divided into 5 equal parts and each part is used as test data, with the remaining 4 parts used for training. Classification accuracy was estimated by taking the average across the 5 parts. The SVM analysis was performed within a 4-mm sphere of all voxels that showed a significant response to at least 1 of the 8 frequency conditions (searchlight procedure; Kriegeskorte et al. 2006). Thus, the classification accuracy for each voxel is based on the activity pattern within the voxel's 34-voxel neighborhood. Group average accuracy was calculated by averaging the co-registered individual classification accuracy volumes. The classification results were visualized on slices through AC, pitched by ∼30° from the line connecting the anterior and posterior commissures to run parallel to the lateral sulcus. In addition, they were also mapped onto the 2-dimensional cortical surface reconstructions for better visualization. Brain surfaces were extracted from the human structural volumes using BrainVoyagerQX (BrainInnovation). Individual brain surfaces were registered to an iterative group average surface (as implemented in BrainVoyager). Surface mapping involves interpolation. The classification analysis was performed on the original volume, rather than the interpolated surface, data, because interpolation introduces correlation between data points, which decreases the power of the classification procedure. Classification accuracies (percent correct classification; the chance level was 12.5%) were transformed to *z*-scores using a binomial distribution and the resulting maps were thresholded at *P* = 0.01 (uncorrected). A cluster-size threshold based on random field theory as implemented in FMRIstat (Worsley et al. 2002) was applied to achieve a threshold of *P* < 0.05, corrected for multiple comparisons. To verify that the classification results were stable and not specific to the particular classification algorithm used, we repeated the analysis with type-1 SVM and *k*-nearest neighbor classifiers. For each classifier, we performed a basic manual grid search for suitable values of its free parameter (*k* in case of the *k*-nearest neighbor classifier and a regularization parameter in case of the SVM). The results were similar between the 2 classifiers and robust across a wide range of parameter values.

The monkey data were analyzed in a similar way as the human data, except that we collapsed multiple frequency conditions into a binary, low versus high, classification problem (with a chance level of 50%). This allowed us to use the same classification problem for all 3 monkeys, 1 of which was presented with only 2 frequency conditions. For Monkey 1, the SVM did not achieve above-chance performance due to the lower number of repetitions relative to the other 2 monkey datasets. For this animal, we used a *k*-nearest neighbor (with *k* = 1) classification algorithm. The *k* = 1 nearest neighbor algorithm uses all training data for classification, rather than only the data at the decision border, as in the case of the SVM. For Monkey 2, we conducted both a 2-category (low vs. high) and a 6-category (corresponding to the 6 presented frequencies; see above) classification analysis to assess the effect of collapsing different frequencies into a 2-category classification problem.

The classification accuracies were used to define “core” AC by thresholding to significantly above-chance classification performance. In order to validate this classification-based method for delineating core AC, the classification results for the monkeys were compared with the results from an independent method proposed by Petkov et al. (2006). They identified macaque core AC as that portion of the STP that (1) responds most strongly to narrowband sounds such as pure tones and (2) contains 3 tonotopic gradients with layouts compatible with those of A1, R, and RT, the locations of which have been verified in previous anatomical studies (Hackett et al. 2001). As the animals were not sacrificed after the fMRI session, no direct cytoarchitectonic parcellation of auditory areas was available.

## Results

### Delineation of Core Auditory Fields

**Figure 1.**
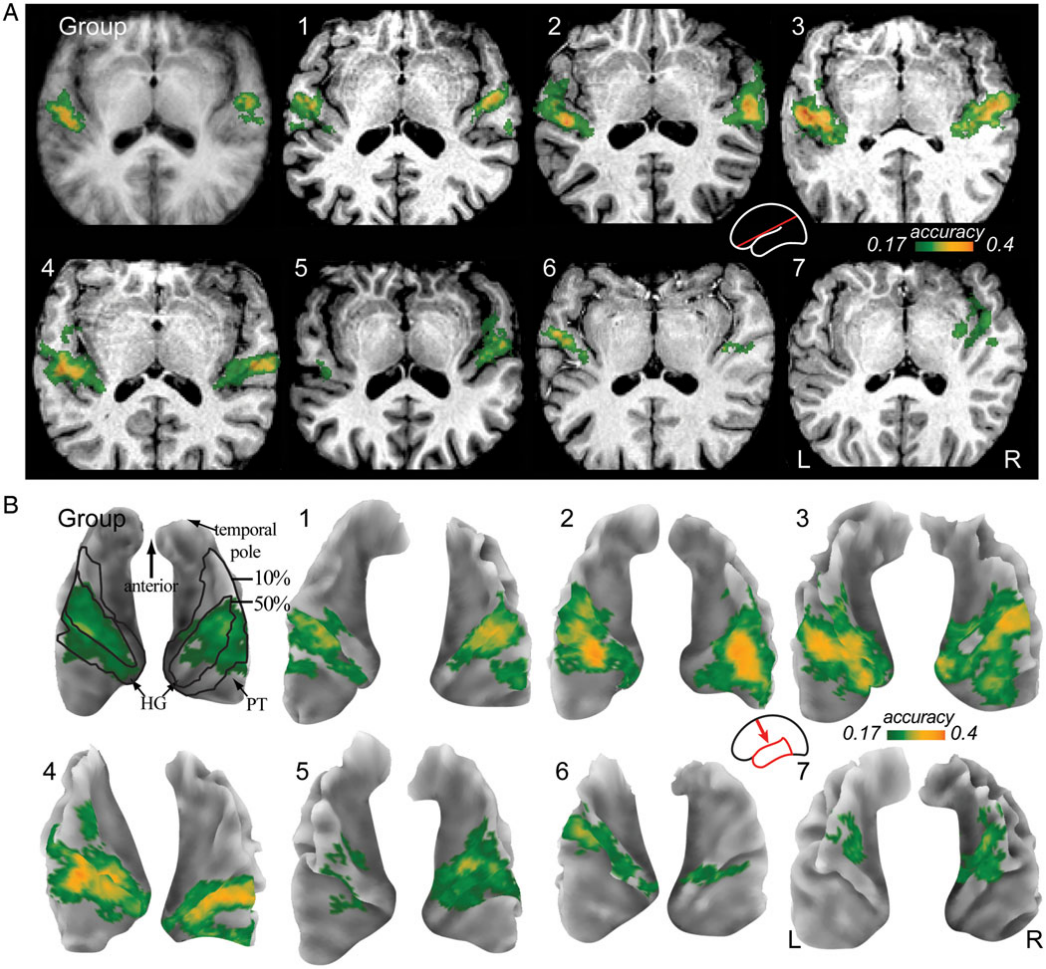
Volume slices (*A*) and surface renderings (*B*) showing the spatial distribution of classification accuracy in AC. Both individual (labeled 1–7) and group average data (labeled Group) are shown. The group data are based on a fixed-effects analysis. The volume slices were oriented to run parallel to the lateral sulcus (see Materials and Methods and inset schematic brain). The cortical surfaces were extracted from the structural volumes; only the upper surfaces of the left and right temporal lobes are shown with HG, PT, and the temporal pole marked in the group average surface (upper left corner in *B*, viewing direction is indicated by the red arrow in the inset schematic brain). The color overlay on each slice or surface shows the performance accuracy of the classification algorithm expressed as proportion of correct classifications and thresholded at *P* = 0.01 in the chance (binomial) distribution. Black lines in the group average surfaces indicate the extent of the 10% and 50% probability maps of cytoarchitectonically defined core AC reported by Morosan et al. (2001). The slice and surface representations are provided to facilitate comparisons with previously published results. In both representations, maxima of classification accuracy are visible on or near HG in all participants.

A crucial prerequisite for parcellating core AC is to delineate core from belt AC. The current approach was based on the hypothesis that, as neurons in core AC respond with greater fidelity and frequency specificity to narrowband sounds than neurons in the auditory belt and parabelt (Rauschecker et al. 1995), fMRI activity in core AC would be more informative about the presented frequency than activity in non-core AC. We tested this hypothesis using pattern classification analysis. Pattern classification analysis assesses the relationship between multi-voxel activity patterns and the stimulus input and is, under certain circumstances, sensitive to features of cortical organization that are below the spatial resolution of the fMRI recording (Boynton 2005; Kamitani and Tong 2005). Here, pattern classification analysis was conducted using a machine-learning approach. We trained a machine-learning algorithm (type-2 SVM) to predict the frequency of each presented stimulus from the activity within small (4-mm) spherical volumes in AC. Prediction accuracy in human datasets reached up to 50% (i.e., the presented frequency was correctly predicted out of a total of 8 possibilities in 50% of cases), which is well above the 12.5% chance level. A confusion matrix analysis between the predicted and actual frequencies revealed that the majority (68%) of misclassifications were between neighboring frequencies. Figure 1 shows the spatial distribution of the classification accuracy on slices through the STP containing HG and on the temporal lobe surfaces. In all 14 hemispheres (7 participants), as well as in the group average data, a contiguous patch of cortical surface on or near HG showed classification accuracy significantly above chance. Some participants showed a second, smaller patch of significant classification accuracy on the PT (6 participants) or planum polare (1 participant). This pattern of results was stable across different classification algorithms (we also tested type-1 SVM and *k*-nearest neighbor algorithms; see Materials and Methods), and a wide range of hyperparameters within each algorithm.

**Figure 2.**
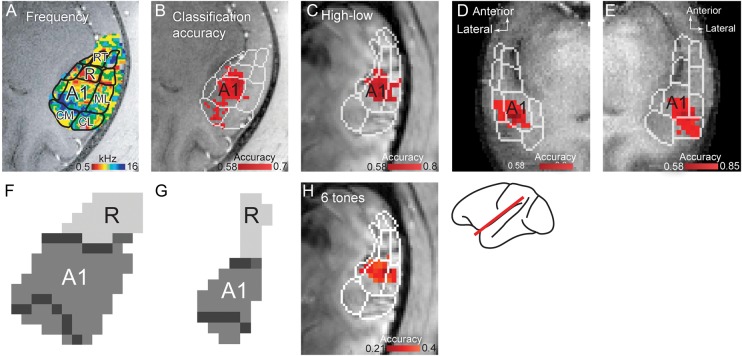
(*A*) A slice through a macaque brain (Monkey 1) parallel to the STP and covering AC. The color overlay shows the sound frequency that evoked the strongest response for each voxel. The black lines delineate different functional fields in AC, identified based on the response amplitude to pure tones and tonotopic gradient reversals. The core fields A1 and R as well as belt fields CM, CL, and AL are labeled. Modified, with permission, from Petkov et al. (2006). (*B*–*E*) The pattern classification results from 3 macaque monkeys (4 hemispheres; *B*, *C*, *E*: right AC of Monkeys 1, 2, and 3; *D*: left AC of Monkey 3; *C*: the classification accuracy when the 6 sound frequency conditions used in Monkey 2 were grouped into high and low frequencies to enable direct comparison with the other monkeys, compare with panel H for results of the 6-frequency classification). The red highlight shows the region where the classification accuracy was significantly above chance (chance levels differed, dependent on the number of frequency conditions). The white lines delineate different AC fields as in *A*. *F* and *G* show automatically determined borders (dark gray) in core fields in the region of interest comprising A1 and R as determined by the manual parcellation shown in *B* and *C*, respectively. The automatically identified border between A1 and R coincided exactly with the manual parcellation. The orientation of the slices in *A*–*E* and *H* is depicted in the inset schematic brain.

In order to test whether the region of significant classification accuracy coincides with core AC, we applied the classification method to fMRI datasets from 3 macaque monkeys (4 hemispheres, because only the right hemisphere was measured in Monkeys 1 and 2). In the monkey data, the location of core AC could be verified using independent functional criteria, as well as existing knowledge from previous anatomical and physiological studies (Petkov et al. 2006). Figure 2 shows the results from pattern classification analysis of the monkey data together with a functional parcellation of each monkey's AC based on response amplitudes to pure tones and frequency gradient reversals (see Materials and Methods). Similar to the results in humans, classification accuracy in a circumscribed region of the monkey AC was greater than that in other regions (the red highlight in Fig. 2*B*–*E* shows significantly above-chance classification accuracy). Importantly, a major portion of this region coincided with the largest core field, A1, in all 4 monkey hemispheres. The second-largest core field, R, was marked in 2 hemispheres, and belt fields CM, CL, and AL were each marked in 1 hemisphere. In contrast, the core field RT was never marked. The monkey data suggest that significant classification accuracy is a useful marker for the core auditory field A1, and, to a lesser degree, also R. From Monkey 2, sufficient data were available to conduct a classification analysis for 6 sound frequency conditions (Fig. 2*H*) in addition to a high- versus low-frequency classification (Fig. 2*C*). Regions of significant classification accuracy were virtually identical in both analyses.

Next, we tested whether the classification accuracy in the human data correlated with the sharpness of the voxel frequency tuning curves or the response amplitude to pure tones, both of which have previously been used as markers for core AC (Wessinger et al. 2001; Petkov et al. 2006; Moerel et al. 2012). We estimated the widths of the voxel frequency tuning curves by measuring the spread around their centroids (see Materials and Methods; Supplementary Fig. 1*A*). For comparison, we also estimated the tuning widths by measuring the full-width-at-half-maximum of bell-shaped (roex) functions fitted to the voxel response curves. The 2 measures of tuning width were moderately correlated (Pearson's *r* = 0.69, *P* < 0.0001). The tuning-curve spreads showed a small, but significant, negative correlation with the classification accuracy (*r* = −0.076, *P* < 0.0001), indicating that spreads were smaller (i.e., tuning curves were sharper) in voxels with higher classification accuracy, as would be expected. The correlation with tuning widths estimated from the fitted roex functions was not significant (*r* = −0.015, *P* = 0.07). To estimate the response amplitude to pure tones, we calculated the maximum of the percentage signal change across the 8 frequencies used in the human measurements (Supplementary Fig. 1*B*), as well as the root-mean-square signal change across all frequencies. Both measures were almost perfectly correlated with each other (*r* > 0.9, *P* < 0.0001), and both showed a small, but significant, correlation with classification accuracy (*r* = 0.08, *P* < 0.0001), indicating that the pure-tone responses were somewhat larger in voxels with higher classification accuracy.

**Figure 3.**
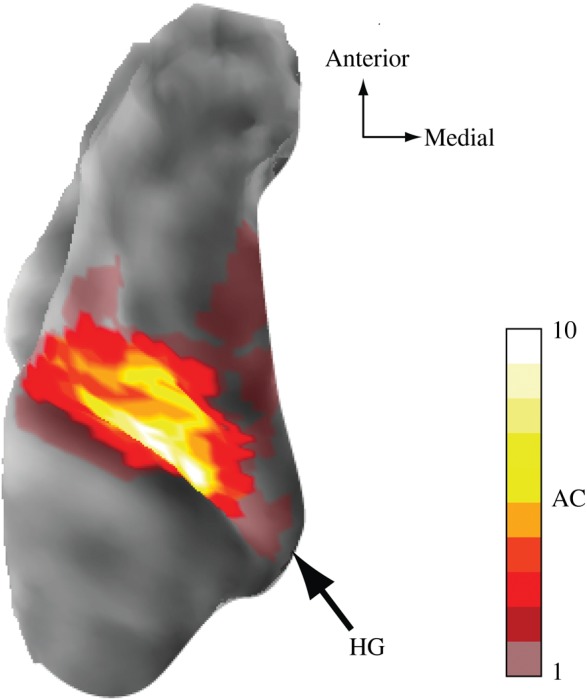
Probability map of estimated core AC. Cortical surfaces of all 14 hemispheres were aligned to the left hemisphere of a target using surface-based alignment of the location of gyri and sulci. The color code indicates the number of hemispheres that contained core AC at a given cortical location. Large overlap of core AC was observed in middle HG.

We computed a probability map of the region of significant classification accuracy across our sample of 14 human hemispheres (Fig. 3). The right hemispheres were flipped to enable this analysis. We found that the overlap of regions of significant classification accuracy in 2 or more hemispheres coincided with the middle part of HG, the same region as identified by cytoarchitectonical parcellations (Morosan et al. 2001; Rademacher et al. 2001). The average overlap of the regions of significant classification accuracy in any 2 hemispheres was 46%. This is similar to the average overlap in functionally defined core AC fields in monkeys (∼50%, see Petkov et al. 2006), suggesting that the locations of core AC fields exhibit a similar degree of inter-individual variability in monkeys and humans.

We also quantified the correspondence between the region of significant classification performance and a previously reported probability map of core AC based on cytoarchitectonic data from 10 postmortem brains (Morosan et al. 2001). For that, we defined 2 regions of interest: one more conservative, corresponding to the 50% probability map of the cytoarchitectonically defined core AC (i.e., the region comprising core AC in at least 50% of brains tested), and the other more liberal, corresponding to the 10% probability map. Outlines of these regions are shown on the group temporal lobe surfaces in Figure 1. We found that the conservative region contained over 90% of the region of significant classification accuracy in the group average data, indicating that there was a good correspondence between the average region of significant classification accuracy and the cytoarchitectonic map of core AC. For individual hemispheres, the correspondence was less good, as would be expected, ranging from as little as 0% to as much as 100%. The liberal region, however, contained 100% of the region of significant classification accuracy not just in the group average data, but in all individual hemispheres bar one (Participant 7, left hemisphere). In this participant, the classification-based estimate of core AC was located more anteriorly and laterally than expected based on the cytoarchitectonic probability map. It is impossible to decide whether the classification-based estimate or the cytoarchitectonic probability map is “incorrect” in Participant 7. The fact that the locations of the classification-based estimates were consistent across the 2 hemispheres would suggest that they are “correct.” At the same time, however, the classification accuracy was generally lower than in other participants, suggesting that the estimates were less reliable.

Rademacher et al. (2001) reported a maximal distance of about 2 cm between the centroids of any 2 cytoarchitectonically defined core AC regions within 27 individuals. Here, we found a very similar maximal distance of 2.3 cm between the centroids of the regions of significant classification accuracy in any 2 of our 14 hemispheres.

### Estimation of Voxel Best Frequencies

The results from the previous section indicate that the region of significant classification accuracy corresponds well with the location of core AC based on cytoarchitectonic criteria in humans and based on independent functional criteria in monkeys. In this section, we describe an automated procedure for measuring tonotopic gradients and finding their reversals in order to delineate different subfields within core AC. The first step in finding tonotopic gradient reversals is to estimate each voxel's preferred, or “best,” frequency. Here, the voxel best frequencies were estimated by calculating the centroid of the voxels' frequency tuning curves (see Materials and Methods). A prerequisite for determining voxel best frequencies is that voxel tuning curves are reliable. We tested reliability by computing the distributions across voxels of the coefficients of correlation between the response curves for the first and second experimental runs. The response curves were highly correlated between the 2 runs in all participants (the distributions of correlation coefficients were skewed toward high values, with a median value of 0.63, on average). Wilcoxon rank sum tests showed that the distributions were significantly different (*P* < 0.0001) from the respective empirical null distributions, estimated by 10 000-fold random sampling (with replacement) from distributions of correlation coefficients between scrambled voxels.

**Figure 4.**
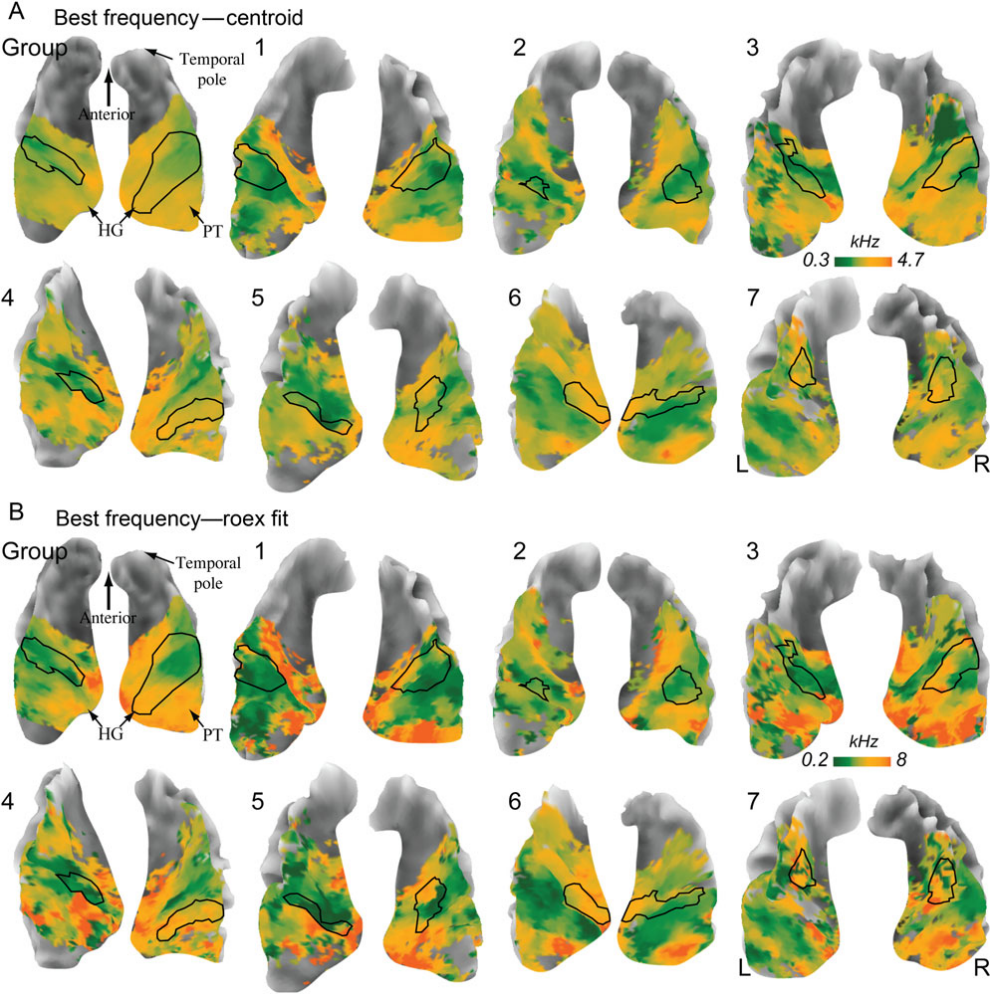
Temporal lobe surfaces with renderings of the voxel best frequencies estimated by calculating the centroids of the voxel tuning curves (*A*), or fitting them with bell-shaped (roex) functions (*B*). In both panels, the outlines of core AC, estimated by thresholding the classification accuracy maps at the significance threshold (see Data Analysis), are marked with black lines.

Based on this result, we then computed best-frequency maps using the centroid method (Fig. 4). For comparison, we also derived the best frequencies from the roex functions fitted to the voxel tuning curves (see Materials and Methods). The best-frequency estimates obtained by the 2 methods were highly correlated (Pearson's *r* = 0.87, *P* < 0.0001); both methods generally yielded a gradual variation in best frequency across the cortical surface. The best frequencies estimated with the centroid method appeared less noisy than those estimated with the function-fit method and thus lent themselves better to extracting best-frequency gradients.

**Figure 5.**
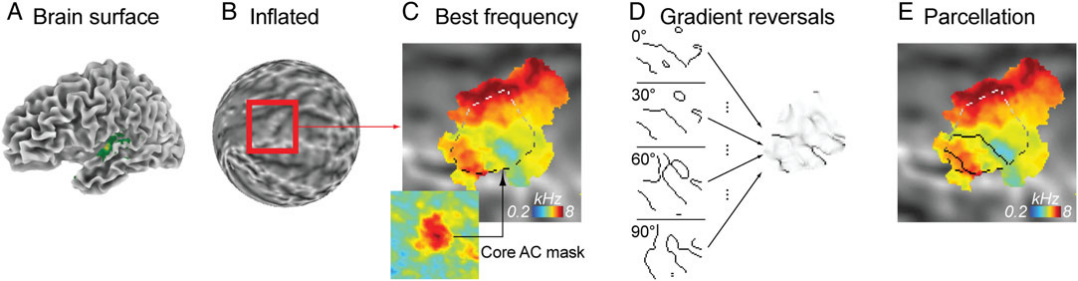
Automatic parcellation of core AC. The extracted individual brain surface (*A*) is inflated (*B*) and a segment covering STP is cut out. (*C*) The best-frequency map is masked with the contiguous region of significant classification performance located closest to HG. This region is taken as estimate of core AC. (*D*) Gradient reversals are computed for all orientations between 0 and 179°. The reversal maps for different orientations are then summed. The values in the resulting map indicate the angle between the adjoining tonotopic gradients. (*E*) Stable reversals indicate the location of subfield borders within core AC.

In order to parcellate core AC into its subdivisions, we used the best-frequency maps to locate reversals in the tonotopic gradients. In the majority of previous tonotopic mapping studies, the orientation of tonotopic gradients was assessed by manually connecting their low- and high-frequency endpoints with straight lines. Only a few studies computed gradient-sign maps (Formisano et al. 2003; Petkov et al. 2006) or analyzed gradient-angle distributions (Langers and van Dijk 2012). Computation of gradient-sign maps involves setting an orientation along which to analyze best-frequency gradients, and then determining, for each voxel, the direction of the best-frequency change (i.e., rising or falling) along that orientation. Edges between positive and negative gradient directions in such maps mark gradient reversals with respect to the pre-set orientation. For instance, if 2 mirror-oriented tonotopic gradients were expected to run parallel to the long (mediolateral) axis of HG, then gradient directions would be determined along that axis of HG. Obvious drawbacks of this method are (1) that it assumes that the gradients adjoining the edge are mirror-symmetric and also (ii) that it requires a prior assumption as to the orientation along which a gradient edge will occur. This is problematic, because the orientations of tonotopic gradients within core AC may vary between participants. A further drawback is that gradient edges are detected along only one, the preset, orientation, and so, no measure of the reliability of the edge locations can be obtained. Our aim was to refine the gradient-sign method in order to eliminate these problems (Fig. 5). For that, we calculated gradient-sign maps, not just for one, but for all possible orientations between 0 and 179°, in 1°-steps. We then marked the locations of gradient reversals for each orientation using an edge detection filter (see Fig. 5*D* for examples). This creates a gradient edge map for each orientation. The edge maps were then summed across all orientations to obtain a composite map of gradient edges representing the number of orientations for which an edge was present at a given location. This composite map allowed us to find the edges with the greatest number of overlapping reversals. Because the orientations were sampled in 1°-steps, the number of reversals represents the angle between the 2 tonotopic gradients adjoining at a given edge location. Thus, the edge with the greatest number of overlapping reversals is also the edge with the largest average angle between adjoining gradients and would thus be presumed to represent the most likely border between fields. In the monkey data, the region of significant classification accuracy overlapped the border between A1 and R in 2 hemispheres, and between A1 and CM and A1 and CL in 1 hemisphere each (see Fig. 2). The borders between A1 and R and between A1 and CM or CL can be distinguished based on the best frequencies of the adjoining voxels: tonotopic gradients share a low-frequency border between A1 and R, but a high-frequency border between A1 and CM or CL. Thus, gradient reversals within regions with low best frequencies likely mark the border between the core fields A1 and R, whereas gradient reversals within regions with high best frequencies likely mark the border between A1 and either of the caudal belt fields CM or CL.

We performed the same automatic detection of area borders in the data from Monkeys 1 and 2 (we could not perform this analysis on Monkey 3 because that dataset contained only 2 sound frequency conditions), using the published delineation of core AC indicated in Figure 2. The automatically determined A1/R borders (Fig. 2*F* and *G*) coincided exactly with the published manual parcellation (Fig. 2*B* and *C*, respectively). In addition, we detected a second, more posterior, gradient reversal in both cases, which may indicate the border between A1 and posterior fields CM or CL. This is consistent with the region of significant pattern classification accuracy, which did not include the posterior portions of manually delineated A1.

**Figure 6.**
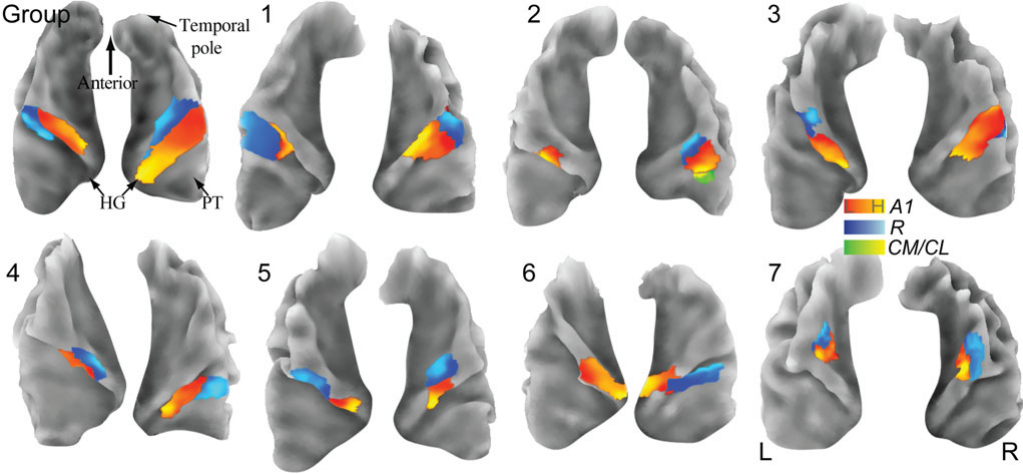
Parcellation results and tonotopic gradients for the human data. All individual hemispheres and the group average data are shown. In most hemispheres (11/14), we found 1 tonotopic gradient reversal with a shared low-frequency border, presumably marking the core fields A1 (red-yellow color gradient, corresponding to low-to-high best frequencies) and R (blue-cyan color gradient). In 1 hemisphere (Participant 2, right hemisphere), we found an additional high-frequency reversal posterior to A1, which probably marks the border between A1 and either of the belt fields CM or CL (green-yellow color gradient). In 2 hemispheres (Participants 2 and 6, left hemispheres), no stable gradient reversals were found in the region of significant classification accuracy.

Figure 6 shows the parcellation results for the human data. In 11 of the 14 hemispheres, the region of significant classification accuracy contained 2 tonotopic gradients with a shared low-frequency endpoint, which presumably marks the border between the core fields A1 and R. One hemisphere showed an additional gradient reversal with a shared high-frequency endpoint, located posterior to the low-frequency reversal. This high-frequency reversal probably marks the border between A1 and either of the caudal belt fields CM or CL. In the remaining 2 hemispheres, the region of significant classification accuracy contained only a single tonotopic gradient and thus no reversals. In these hemispheres, the gradient orientation was consistent with A1.

**Figure 7.**
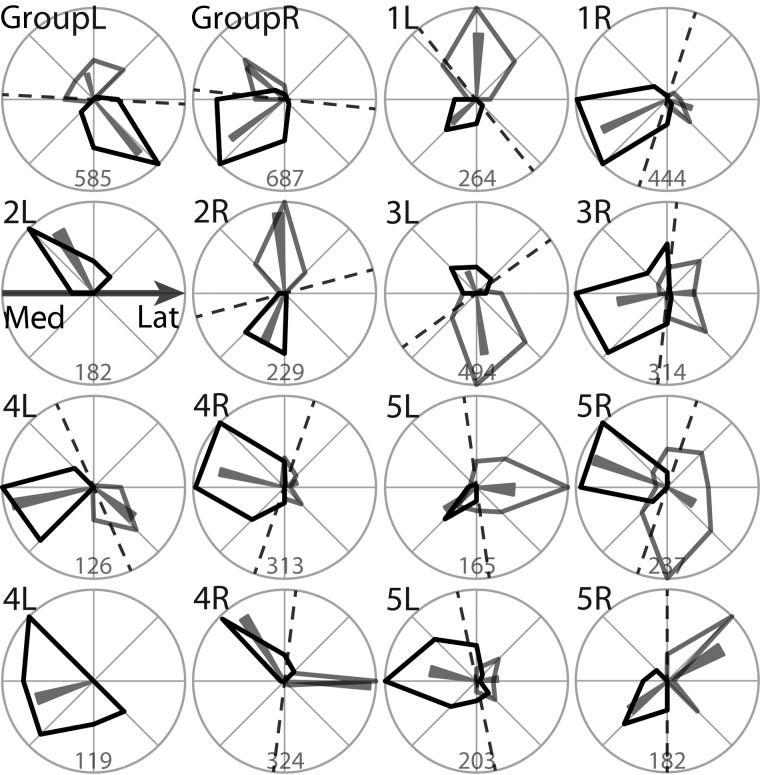
Circular histograms of tonotopic gradient direction in A1 (black polygons) and R (gray polygons) in left (L) and right (R) hemispheres of the group data and Participants 1–7. Directions are relative to the orientation of HG, whose medial-to-lateral axis corresponds to the horizontal axis in each plot (indicated by the black arrow in plot 2L). Directions were binned every 45° (light-gray circle sections) for plotting. Mean directions and 95% confidence intervals are indicated by small gray triangles. Numbers at the bottom of each graph indicate the upper axis limit of the histogram as number of vertices. Dashed black lines show the orientation of the border between A1 and R, as identified by the gradient reversal analysis.

We measured the direction of the tonotopic gradient with respect to the orientation of the long (mediolateral) axis of HG at each vertex on the flattened cortical surfaces in A1 and R in each participant and the group map (Fig. 7). Mean angles ranged from almost parallel (1°) to almost perpendicular (88°) with a mean of 34° (interquartile range 30°). The mean angle in the group map was 50°. In individual hemispheres, 19 out of 26 of the mean directions were below 45°. This proportion and larger ones are unlikely to occur under the assumption of an equal distribution of angles within the measured range (under 2% of 100 000 bootstrap samples), suggesting that mean gradients tend to align with the long axis of HG. We also examined the orientation of the border between A1 and R identified by the gradient reversal analysis. Although these edges are not straight, a representative angle can be computed as the complement to 90° of the direction for which the edge map correlates maximally with the sum of edge maps across all directions (for instance, in Fig. 5*D*, the gradient reversals at 30° correlate better with the summed map than the ones at 90°). In the group map, the border between A1 and R so computed ran almost parallel to HG (3° on the left and 6° on the right side, Fig. 7). In the majority of the individual hemispheres, these angles were above 70°, that is, almost perpendicular to HG. The mean angle was 67° with an interquartile range of 23° and a range of 15–90°.

## Discussion

This study aimed to map core AC in humans using fMRI. In contrast to previous tonotopy studies, we used multi-voxel pattern classification of sound frequency to distinguish core from belt AC, and we assessed tonotopic gradients and gradient reversals using an automated procedure unbiased by prior assumptions to delineate subfields within core AC. We limited the tonotopic gradient analysis to the region identified as core by the pattern classification analysis. This avoids the risk of constructing erroneous tonotopic gradients across borders between core and belt fields (see also Dick et al. 2012; Moerel et al. 2012). We validate our method with fMRI tonotopy datasets from 3 macaque monkeys. Our results challenge a model of core AC organization in humans proposed by several recent fMRI tonotopy studies (Humphries et al. 2010; Da Costa et al. 2011; Langers and van Dijk 2012) but are consistent with previous cytoarchitectonic (Morosan et al. 2001) and myeloarchitectonic (Sigalovsky et al. 2006; Dick et al. 2012) measurements of human core AC and accord with a recently proposed unified model of the primate primary AC (Baumann et al. 2013). Unlike the myelination measurements, the pattern classification method involves no specialized imaging sequences or analysis techniques and can also be applied to already existing tonotopy datasets. The current results suggest that, at least at 3 T, where the current human data were acquired, the pattern classification approach may be more sensitive than the previously used criterion of sharper frequency selectivity in core AC (Moerel et al. 2012; see below).

### Location of Core Auditory Fields

Frequency classification accuracy peaked on or near HG in all of the 14 human hemispheres measured in this study. In the 4 macaque monkey hemispheres, the region of significant classification accuracy consistently overlapped the largest core field A1 and, in 2 hemispheres, also the smaller core field R. In 2 hemispheres, the significant classification region also overlapped the caudal belt fields CM or CL. This is consistent with previous findings of tonotopic organization in these areas in primates (Kaas and Hackett 1998; Petkov et al. 2006). Physiological studies have shown that frequency selectivity is lower in belt than in core fields (Rauschecker et al. 1995). This may explain why significant classification accuracy in the caudal belt was observed in only 2 of our 4 macaque hemispheres. Note, however, that the parcellation of the monkey AC proposed by Petkov et al. (2006), against which the current classification results were compared, was not verified by postmortem anatomical analysis and may thus itself exhibit inaccuracies. That study also reported considerable variability in the tonotopic maps within and between animals. Irrespective, there is a clear convergence of human and macaque results in that the region of significant classification accuracy corresponded roughly to the expected location of core AC and, in the majority of cases, contained 2 tonotopic maps with orientations consistent with the 2 largest core fields, A1 and R.

The classification results were robust across different classification algorithms and parameters, suggesting that the frequency classification method is a useful *in vivo* marker of core auditory field A1 and, to a lesser extent, R in individual brains. We would expect that the region of significant classification accuracy would also be robust against changes in the stimulation paradigm or parameters. It has been shown that the size of the sustained response (SR) to longer stimulus sequences (>6 s) is affected by the stimulus rate, with faster rates yielding a larger SR in primary fields, and slower rates yielding a larger SR in nonprimary fields (Giraud et al. 2000; Harms and Melcher 2002; Seifritz et al. 2002). The current study used relatively short stimulus sequences (4 s), and so, the measured activity would be expected to represent the onset response to the sequences, which is relatively little affected by the stimulus rate (e.g., Harms and Melcher 2002). When using longer sequences, an intermediate stimulus rate should be used in order to create balanced activity across both primary and non-primary fields.

In the human participants, classification accuracy correlated weakly with the sharpness of the frequency tuning curves and with the overall response amplitude to pure tones. This was expected because neurons that are more sharply tuned in frequency and thus more strongly driven by narrowband sounds such as pure tones should also be more informative about the presented frequency. Sharpness of frequency tuning and response amplitude to pure tones and other narrowband sounds have been used in previous studies as criterion to delineate core AC in humans (Wessinger et al. 2001; Chevillet et al. 2011). The classification method might be expected to be more sensitive than the sharpness-of-tuning criterion, because classification analysis of multi-voxel activation patterns has been shown to be less susceptible than single-voxel-based analyses to limitations in the measurement spatial resolution (determined by the voxel size and the spatial spread of the blood oxygen level-dependent effect). Multi-voxel pattern analysis can be used to extract information encoded at a somewhat smaller scale than the voxel dimensions and does not require measurable tuning at the voxel level (Boynton 2005; Kamitani and Tong 2005). The criterion of response amplitude to narrowband sounds is premised on the assumption that core and belt AC differ in their frequency tuning properties, but not in their overall sensitivity to sound. Given that response amplitudes also depend on stimulus parameters other than frequency composition (e.g., stimulus rate; see above), this assumption cannot be generally valid. Moreover, due to nonlinearities in the blood oxygenation level-dependent effect (Liu et al. 2010), large differences in the neural response amplitude between core and belt AC might translate to only small differences in the amplitude of the fMRI signal.

The results from the classification method show a considerable degree of variability in the location of human core AC across our sample of 14 hemispheres. Much of this variability is due to macroanatomical variability. A probability map of the region of significant classification accuracy, calculated using surface-based alignment of the temporal lobes across participants and hemispheres, showed a good degree of overlap of core AC around middle HG. The location and variability of the region was consistent with the previous cytoarchitectonic (Morosan et al. 2001; Rademacher et al. 2001) and myelination measurements (Sigalovsky et al. 2006; Dick et al. 2012). The group average region of significant classification accuracy was for the most part (90%) contained within the cytoarchitectonic 50% probability map of Morosan *P et al.* (2001). We do not have myelination data for our participants (the data acquisition predated those reports) and thus cannot conduct a direct within-participants comparison of myelination and classification accuracy. However, at the group level, the region identified by the classification method was largely congruent with the region that exhibited high myelination in the study by Dick et al. (2012). Both sets of results suggest that core AC may extend less widely than assumed in several recent human tonotopy studies (Humphries et al. 2010; Da Costa et al. 2011; Langers and van Dijk 2012). These studies assumed that core AC encompasses the entire HG and also some of the regions anterior and posterior to HG. In contrast, both our classification results and the myelination results by Dick et al. (2012) suggest that, on average across participants, core AC encompasses only a relatively small, circumscribed region on middle HG.

The convergence between independent (functional and anatomical) localizers bolsters confidence in their validity and highlights the potential benefit of combining them in 1 experiment. The method proposed by Dick and colleagues and our classification method both require thresholding (on the myelin measure, R_1_, and on classification accuracy, respectively) to delineate core fields, and combining information, for instance in a Bayesian framework, may help to reduce potential bias from threshold selection.

### Orientation of Tonotopic Gradients

The orientation of the tonotopic gradients within the core AC region in individual hemispheres varied between 1 and 88° relative to the long axis of HG, with an average of 34° and an interquartile range of 30°. The automatically identified border between the presumed core fields A1 and R was oriented almost perpendicular to HG in most hemispheres, with a mean angle of 67°, a range of 15–90°, and an interquartile range of 23°. In the monkey data, the border identified by our automatic method coincided exactly with the previously published manual delineation. The method proposed here to identify reliable gradient reversals is robust against variation in gradient orientation, because it is not dependent on any assumption of the orientation of tonotopic gradients with respect to HG. The centroid method for estimating voxel best frequencies helped to reduce noise in the best-frequency maps, which makes the extraction of gradient reversals more reliable. Previous studies have demonstrated good repeatability of tonotopic mapping with fMRI across time and stimulus types (Da Costa et al. 2011; Dick et al. 2012; Moerel et al. 2012). Similarly, the current data showed good repeatability of voxel frequency tuning curves across measurement runs. We therefore think that the observed variability in the tonotopic gradient orientations represent, at least partly, true differences between individuals and hemispheres. This is consistent with physiological data: in the earliest tonotopic mappings of animal AC, Merzenich and colleagues report a significant variation in the location of core AC and in the orientation of best-frequency gradients in core AC in macaque monkeys (Merzenich and Brugge 1973), squirrels (Merzenich et al. 1976), and cats (Merzenich et al. 1975). In the description of the results on cat AC, the authors explicitly stated that it was necessary to consider each cat individually in order to arrive at a coherent model of tonotopic organization. Variability in the tonotopic gradients has also been reported in fMRI studies in macaques (Petkov et al. 2006; Baumann et al. 2010; Tanji et al. 2010). Although often de-emphasized, considerable inter-individual variation is also evident in human tonotopy studies that show individual data (Schönwiesner et al. 2002; Formisano et al. 2003; Talavage et al. 2004; Humphries et al. 2010; Da Costa et al. 2011; Langers and van Dijk 2012; Dick et al. 2012; Moerel et al. 2012), and in macaque fMRI tonotopic maps (Petkov et al. 2006).

The general pattern of frequency preference across the STP observed in our group data is consistent with the results found in previous studies (Talavage et al. 2004; Humphries et al. 2010; Da Costa et al. 2011; Langers and van Dijk 2012; Dick et al. 2012; Moerel et al. 2012). All these studies show a broad region of low best frequencies on the anterolateral crown of HG, and a wide collar of high best frequencies around the posteromedial end of HG and extending both anteriorly and posteriorly along HG. However, different studies have interpreted this pattern differently depending on whether or not they used an independent criterion to delineate core AC. The studies that did not use an independent criterion (Humphries et al. 2010; Da Costa et al. 2011; Langers and van Dijk 2012) proposed a model of human core AC in which the core fields A1 and R run all the way along the anterior and posterior banks of HG, sharing a border roughly parallel to its long axis (see, for instance, Humphries et al. 2010, Fig. 9). The assumption is that the tonotopic gradients in both A1 and R run quasi-perpendicular to HG, connecting the low-frequency region on its anterolateral crown to the high-frequency strips anterior and posterior to HG, respectively. However, our classification results suggest that these low- and high-frequency regions do not represent corresponding endpoints of tonotopic gradients. In particular, our results, together with myeloarchitectonic mapping (Dick et al. 2012) and other functional criteria (Moerel et al. 2012), suggest that only the best-frequency progression within a relatively small, circumscribed region around the middle part of HG represents a coherent tonotopic gradient, presumably the gradient of area A1. A quasi-perpendicular layout would also be in direct disagreement with the cytoarchitectonic parcellation of human core AC by Morosan et al. (2001). These authors found 3 subfields (which they referred to as Te1.0, Te1.1, and Te1.2), stacked along HG, and with borders running perpendicular, rather than parallel, to its long axis. The best-frequency progression is variable across individual hemispheres but runs approximately parallel to the long axis of HG in the majority of hemispheres tested here (8/14). This is consistent with several earlier human tonotopy studies (Talavage et al. 2000, 2004; Formisano et al. 2003; Upadhyay et al. 2007), which have also found a low- to high-frequency progression from anterolateral-to-posteromedial HG.

In addition to A1 and R, a third core subfield, RT, has been demonstrated in several monkey species. There is growing evidence for the existence of RT in humans. Morosan et al. (2001) reported 3 primary-like fields in human postmortem brains, but the correspondence between these fields and functionally defined A1, R, and RT, is unclear. While the earlier human tonotopy studies did not find a second tonotopic gradient reversal (Talavage et al. 2000; Schönwiesner et al. 2002; Formisano et al. 2003; Talavage et al. 2004), some of the more recent studies reported a second reversal, located anterior or lateral to the first reversal (Woods et al. 2009; Humphries et al. 2010; Moerel et al. 2012). The second reversal might mark the R–RT border. RT is small and exhibits a less consistent tonotopic organization than A1 and R (Petkov et al. 2006). This may be why RT failed to yield significant frequency classification accuracy in our monkey datasets. Alternatively, the tonotopic map of RT may be incomplete in humans (i.e., only cover a part of the frequency range). An inconsistent or incomplete tonotopic organization of RT may mean that a definitive marker of the entire core AC may have to comprise a combination of functional, anatomical, and perhaps connectivity criteria.

In this study, we could not extend the tonotopic mapping beyond core AC, because we were unable to distinguish between belt and parabelt fields. At present, the separation of belt from parabelt AC would have to be inferred from the known organization of the non-human primate AC and would thus be highly speculative. Moreover, tonotopic gradients would be expected to be less clear outside of core AC. However, at least belt fields would be expected to exhibit tonotopic organization with best-frequency gradients collinear to those of the adjacent core areas. It may thus be hoped that identification of core AC as in the current study will enable delineation of at least some of the adjoining fields.

## Supplementary Material

Supplementary can be found at: http://www.cercor.oxfordjournals.org/.

## Funding

This work was supported by the Discipline Bridging Award of the University of Nottingham, UK. MS was supported by the German Academy of Sciences during part of this work, PD was supported by the Volkswagen Foundation, KK was supported by the Medical Research Council (UK), and CP was supported by the Wellcome Trust (UK). Funding to pay the Open Access publication charges for this article was provided by the Medical Research Council (UK).

## Supplementary Material

Supplementary Data
