# Supplementary material for: Parcellation of Human and Monkey Core Auditory Cortex with fMRI Pattern Classification and Objective Detection of Tonotopic Gradient Reversals
Source: Cereb Cortex. 2014 Jun 5;25(10):3278–89. doi: 10.1093/cercor/bhu124 (PMC4585487; doi:10.1093/cercor/bhu124)
Supplement: Supplementary Data [file supp_25_10_3278__index.html]

Parcellation of Human and Monkey Core Auditory Cortex with fMRI Pattern Classification and Objective Detection of Tonotopic Gradient Reversals — Parcellation of Human and Monkey Core Auditory Cortex with fMRI Pattern Classification and Objective Detection of Tonotopic Gradient Reversals — Supplementary Data 

# Parcellation of Human and Monkey Core Auditory Cortex with fMRI Pattern Classification and Objective Detection of Tonotopic Gradient Reversals

## Supplementary Data

Supplementary Data

**Files in this Data Supplement:**

- Supplementary Data - Docx file
