# Supplementary material for: Parcellation of Human and Monkey Core Auditory Cortex with fMRI Pattern Classification and Objective Detection of Tonotopic Gradient Reversals
Source: Cereb Cortex. 2014 Jun 5;25(10):3278–89. doi: 10.1093/cercor/bhu124 (PMC4585487; doi:10.1093/cercor/bhu124)
Supplement: Supplementary Data [file supp_bhu124_bhu124supp.docx]

**Supplemental Figure**

**
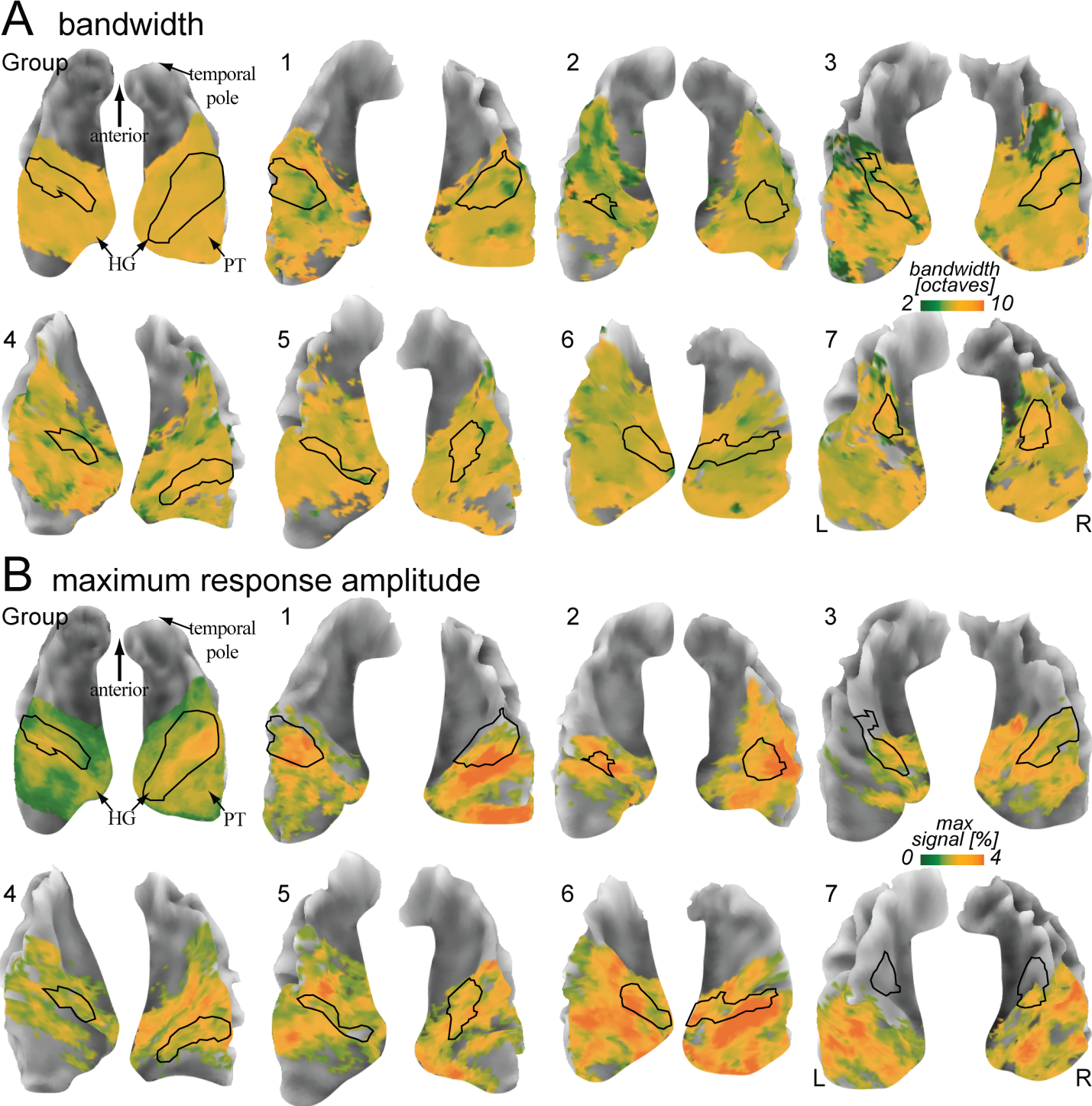
**

**Figure S2:** A) Temporal lobe surfaces as in Fig. 1, with renderings of the spreads of the voxel frequency tuning curves, expressed in octaves. Both individual (1-7) and group average data (Group; based on fixed-effects analysis) are shown. B) The same surfaces as in panel A, but with renderings of the maximal response across all pure-tone frequencies, expressed in percent signal change. In both panels, the outlines of core AC, estimated on the basis of significant classification accuracy, are marked with black lines.
